# A Case of Lymphangioma of the Calf Region: Imaging Spectrum With Histopathological Correlation

**DOI:** 10.7759/cureus.48818

**Published:** 2023-11-14

**Authors:** Sidharth Gupta, Suresh Phatak, Prashant Onkar, Ashish N Ambhore, Kajal Mitra

**Affiliations:** 1 Department of Radiodiagnosis, Narendra Kumar Prasadrao (NKP) Salve Institute of Medical Sciences and Research Centre and Lata Mangeshkar Hospital, Nagpur, IND

**Keywords:** colored flow doppler ultrasound, muskuloskeletal mri, lymphatic malformation, unilateral leg swelling, cystic lymphangioma

## Abstract

Lymphangioma, also known as cystic hygroma are benign malformations arising from abnormal development of the lymphatic system. Most often these lesions are found in the pediatric population, having a predilection for the neck/axilla, and are less common in extremities. Symptoms can vary based on size and location. Treatment is not usually indicated until they start impacting life due to deformity or symptoms such as pain, paraesthesia, etc. Here, we report a case report of lymphangioma located in the calf region of the right lower limb presenting in adult age.

## Introduction

After venous malformations, lymphatic malformations rank second in frequency [1]. Lymphangiomas are rare congenital malformations of the lymphatic system arising due to the failure of lymphatic channels to communicate with the larger lymphatic vessels leading to the formation of cystic spaces filled with lymphatic fluid. Lymphatic malformations can be classified into various types including microcystic, macrocystic, or mixed types. This classification depends on the size of the cystic lesion (less than or greater than 2 cm). Macrocystic malformations are also known as cystic lymphangiomas/cystic hygromas which consist of large cystic spaces having collagen and smooth muscle. Microcystic malformations are a common occurrence in children and present as swellings in the subcutaneous plane. Mostly presenting in childhood, cases in adults are rare [2].

## Case presentation

A 31-year-old lady presented with a progressively enlarging mass in her right lower limb over the calf from the last 20 years. Although initially asymptomatic, she gradually developed a sensation of heaviness, discomfort, and pain, especially after prolonged periods of standing.

**Figure 1:**
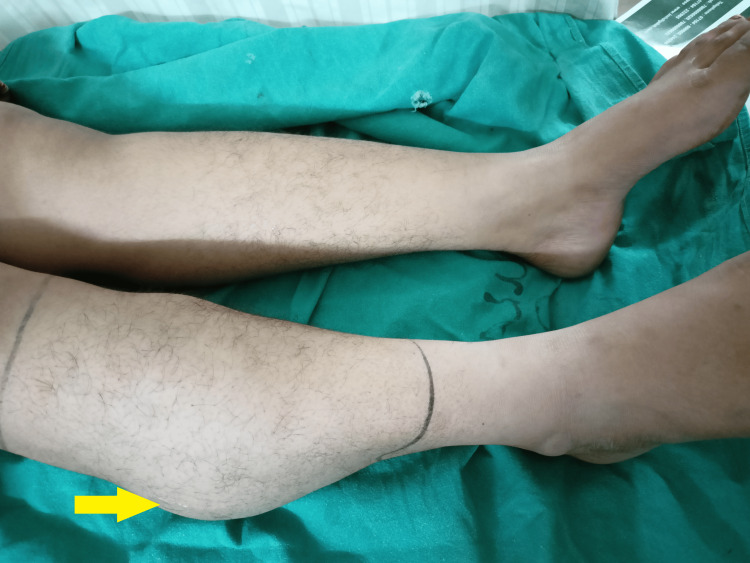
Clinical photograph showing right lower limb swelling over the calf region (indicated by a yellow arrow)

On physical examination, a soft, non-tender, fluctuant mass measuring around 11 cm in length was noted over the right calf region. The overlying skin appeared normal without any signs of inflammation (Figure 1).

**Figure 2:**
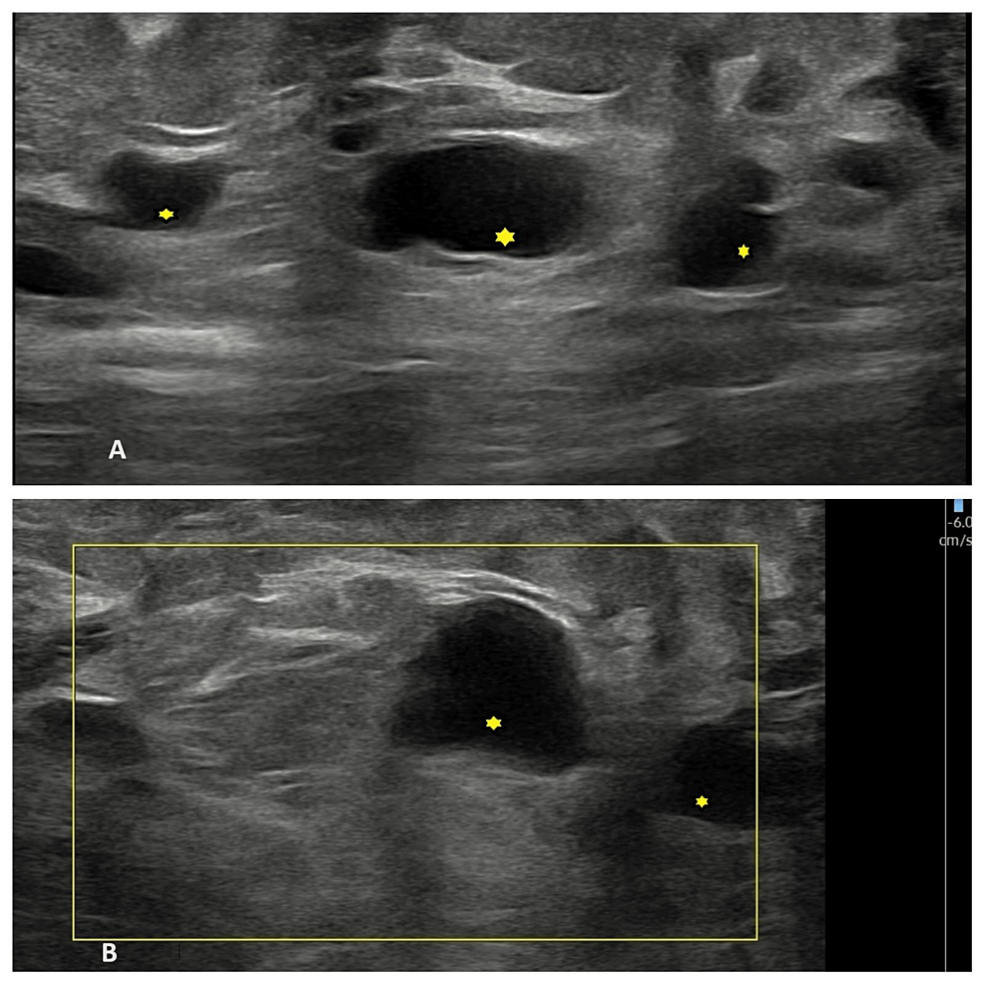
(A): B-Mode ultrasound of the right calf region showing an anechoic multilocular cystic lesion in the subcutaneous plane (indicated by yellow stars). (B): On color Doppler imaging, no vascularity is noted within these cystic spaces (indicated by yellow stars)

Routine blood investigations and chest radiographs were within normal limits. The patient was subsequently referred for an ultrasound and MRI examination. On ultrasound examination, an anechoic multilocular cystic lesion was noted in the subcutaneous plane that did not show flow on colour Doppler (Figure 2).

On MRI examination, a large ill-defined multiloculated cystic lesion was noted in the subcutaneous plane in the anterolateral and posterior aspect of the right leg over the calf region, showing maintained fat planes with adjacent muscles and adjacent fat hypertrophy.

**Figure 3:**
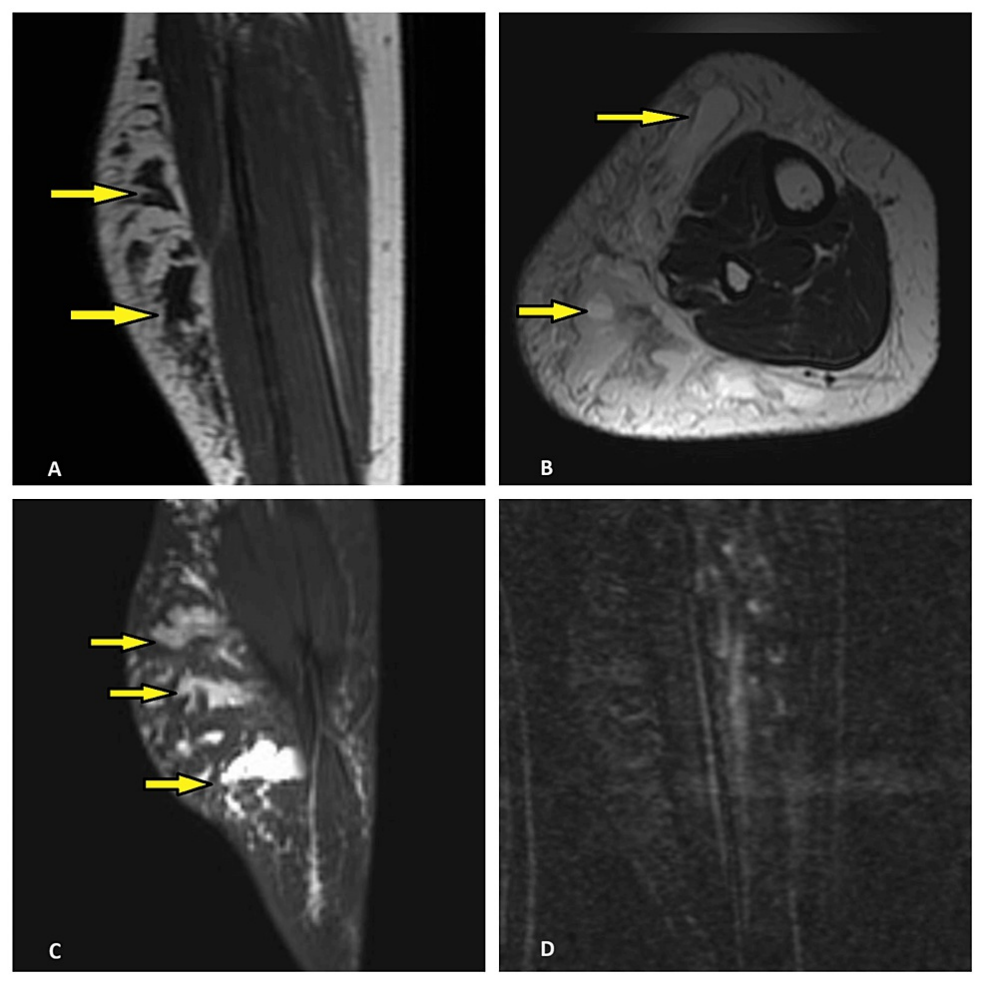
(A): Sagittal T1-weighted MRI image showing large, ill-defined multiloculated cystic lesion (indicated by yellow arrows) in the subcutaneous plane in the posterior aspect of right leg appearing hypointense on T1-weighted image. (B): Axial T2-weighted image showing multiloculated cystic lesion (indicated in yellow arrows) in the anterolateral and posterior aspect appearing hyperintense on T2-weighted image. (C): The lesion appears hyperintense in the sagittal STIR image (indicated by yellow arrows). (D): On post-contrast study, no enhancement is noted STIR: Short tau inversion recovery

This lesion appeared hypointense on the T1-weighted image, hyperintense on the T2-weighted image, and short tau inversion recovery. In the post-contrast study, no enhancement was noted (Figure 3).

**Figure 4:**
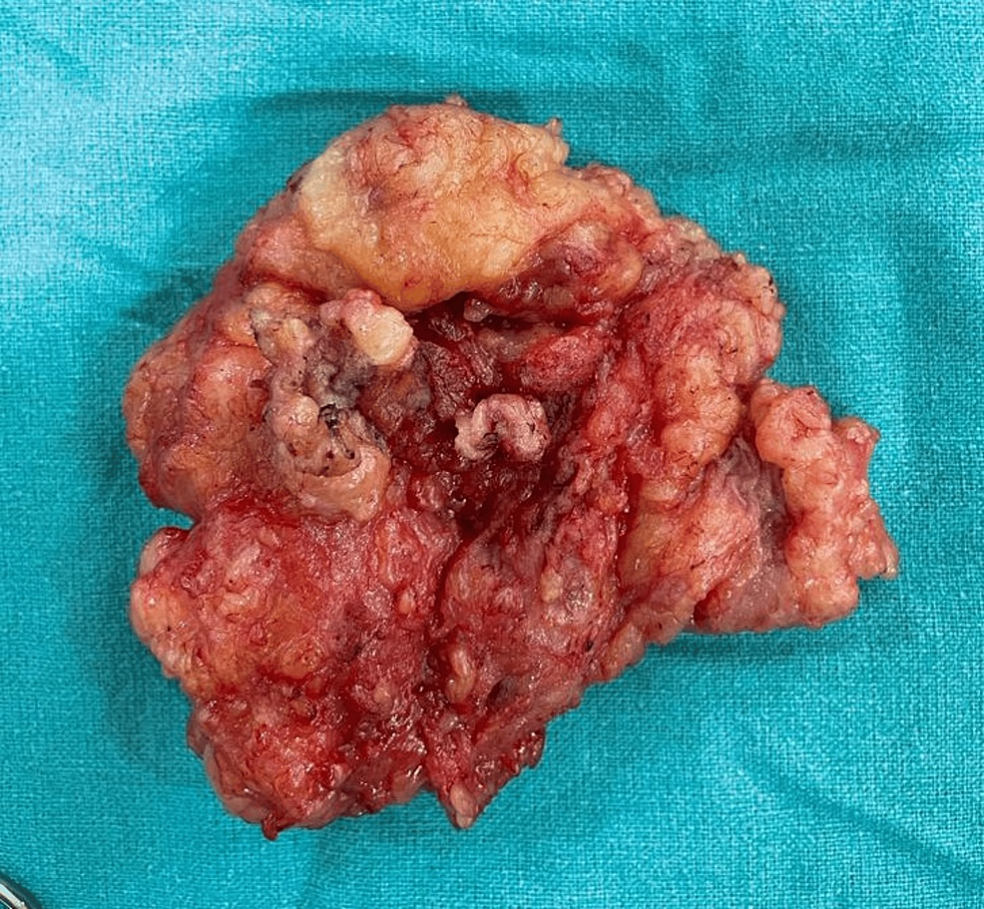
Post-operative specimen showing lobulated, reddish mass having an admixture of solid and spongy areas

Based on our imaging findings, lymphangioma was suggested as the probable diagnosis, and the patient was subsequently operated on where the post-operative specimen showed a lobulated, reddish mass having an admixture of solid and spongy areas (Figure 4).

**Figure 5:**
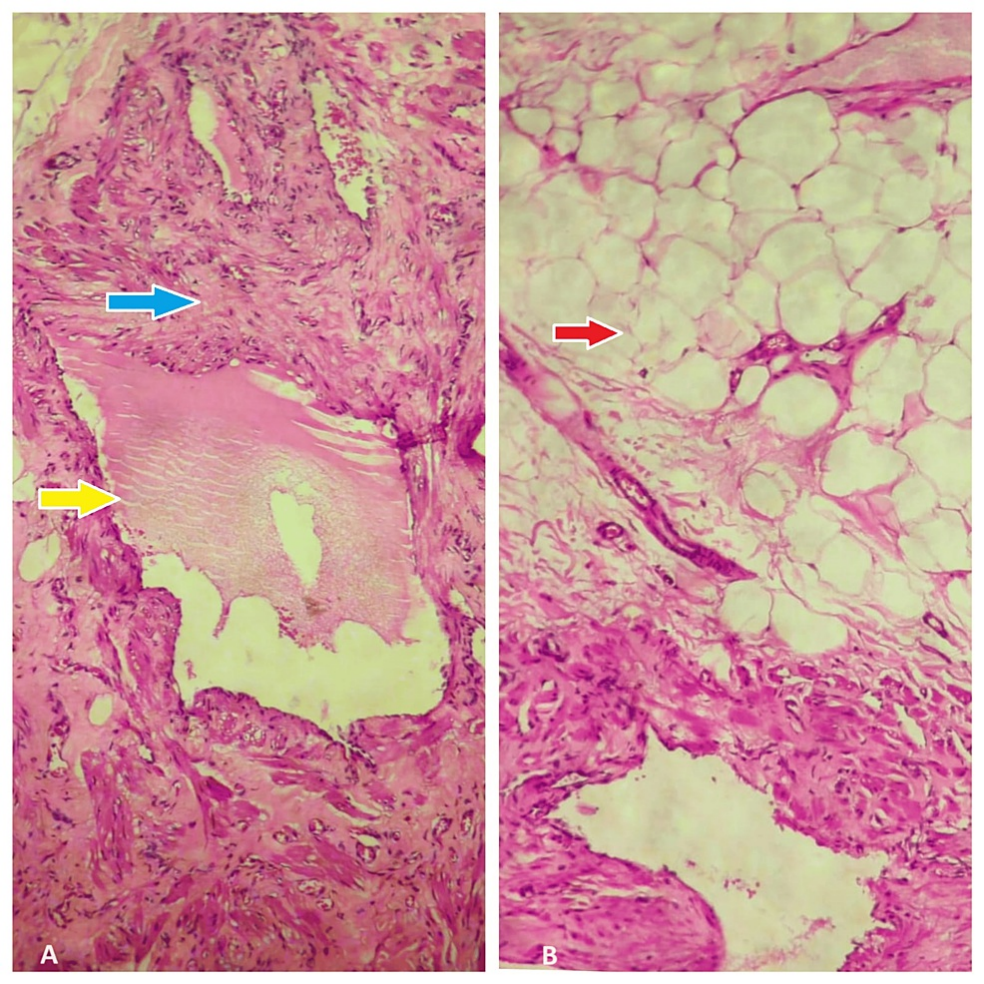
Histopathology showing cystically dilated lymphatic spaces lined by flattened epithelial cells (indicated by a yellow arrow). Surrounding fibromuscular (indicated by a blue arrow) and fibroadipose (indicated by a red arrow) tissue is seen

Histopathology of the tissue subsequently revealed irregular fragments of tissue composed of cystically dilated lymphatic spaces containing lymphoid aggregates lined by flattened epithelial cells. Surrounding fibroadipose and fibromuscular tissue was seen (Figure 5). Thus, the diagnosis of cystic lymphangioma was established.

## Discussion

Cystic lymphangiomas are circumscribed deep lymphangiomas that are usually located in the cervicofacial and axillary region and less commonly in the retroperitoneum, mesentery, and mediastinal region [3]. Occurrence in limbs is rare [4]. The role of certain regulatory genes like vascular endothelial growth factor receptors 3 and 2 that are involved in the growth of lymphatics has been implicated in pathogenesis [5].

Ultrasound helps in the evaluation of these lesions and shows multilocular cystic masses with anechoic or echogenic content which depends on whether lymphatic fluid is infected/hemorrhagic/hyperlipidic [6]. On CT, the appearance can be highly variable, ranging from purely cystic lesions to heterogeneous lesions consisting of both cystic and solid components. On post-intravenous contrast administration, no enhancement is noted. However, CT helps in differentiating this lesion from hemangiomas where calcified phleboliths can be noted. On MRI, these malformations appear as multiloculated, cystic masses that may also invade the adjacent tissues, causing hypertrophy of the affected body part sometimes. These cysts usually have a low signal on a T1-weighted image and a high signal on a T2-weighted image [7]. Heterogeneous signals can be seen within these cysts due to the presence of proteinaceous/hemorrhagic content. Enhancement is usually not present, although the septa may show uptake of contrast as they are vascularized. MRI helps in further evaluation of the lesion by allowing for a more accurate evaluation of the tumor and its relationship to adjacent structures, thus proving valuable to surgeons [8,9]. Magnetic resonance (MR) lymphangiography is being increasingly used in place of traditional invasive lymphangiography and can be performed with or without contrast administration [10]. The lymphatic system being an essential component of the circulatory system is altered by various pathological processes. Dynamic contrast material-enhanced MR lymphangiography evaluating the anatomy of lymphatic malformations [11].

Cystic lesions of the extremities are a common presentation in clinical practice. These lesions may be true cystics such as ganglia/bursae/synovial cysts that exhibit bright signals on a T2-weighted image. However, many solid benign lesions such as myxomas and peripheral nerve sheath tumors or malignant lesions like pleomorphic sarcomas, synovial sarcomas, extra-skeletal myxoid chondrosarcomas, etc. may also present with T2 hyperintensity. Inflammations or hemorrhages in cystic lesions may result in complex appearances [7].

Lymphangiomas are challenging to manage due to their complex anatomical characteristics and potential for recurrence [12]. Surgical excision, laser therapy, and sclerotherapy are common treatment options. Management depends on multiple variables like the extent, location, symptoms of the patient, and their overall health. Multidisciplinary collaboration and personalized treatment planning are essential for achieving successful outcomes and minimizing the risk of recurrence. Sclerotherapy which works by obliterating the lumen [13] appears to be a viable option for the management of lymphangiomas, offering a significant reduction in size and improvement in symptoms.

## Conclusions

Lymphangiomas of the lower limb are rare tumors. Ultrasound and MRI are useful imaging modalities for accurate diagnosis and patient management.
